# Relationship of Retinal Vessel Caliber with Age-Related Macular Degeneration

**DOI:** 10.1155/2022/8210599

**Published:** 2022-07-31

**Authors:** Sara Toulouie, Sean Chang, Julia Pan, Kiersten Snyder, Glenn Yiu

**Affiliations:** ^1^Department of Ophthalmology & Vision Science, University of California, Davis, Sacramento, CA, USA; ^2^California Northstate University, College of Medicine, Elk Grove, CA, USA

## Abstract

**Purpose:**

Evaluate the relationship between retinal vascular caliber and age-related macular degeneration (AMD) severity or progression.

**Methods:**

A retrospective secondary analysis of 1172 fundus photographs and clinical data from the prospective Age-Related Eye Disease Study (AREDS). Central retinal artery equivalent (CRAE), central retinal vein equivalent (CRVE), and arteriole-to-venule ratio (AVR) were measured using the Parr–Hubbard–Knudtson formula. Univariate and multivariate regressions were used to determine the association of CRAE, CRVE, and AVR with age, sex, smoking status, presence of cilioretinal artery, and AMD severity at baseline and 5 years using the 9-step AMD severity score.

**Results:**

Only CRAE and CRVE were higher in men (*P* < 0.001), current smokers (*P* < 0.001), and the eyes with a cilioretinal artery (*P*=0.009 − 0.043). AMD severity was greater in older patients (*P*=0.001), current smokers (*P*=0.012), the eyes without a cilioretinal artery (*P*=0.001), and lower AVR (*P*=0.034) on multivariate regression but was not influenced by CRAE or CRVE (*P*=0.240 − 0.500). Choroidal neovascularization (CNV) presence was associated with older age (*P*=0.003) and absence of a cilioretinal artery (*P*=0.009), while central geographic atrophy (CGA) was associated with narrower CRAE (*P*=0.002) and possibly AVR (*P*=0.046). None of the retinal vessel parameters were predictive of AMD severity score or new onset of CNV or CGA at 5 years.

**Conclusion:**

A lower arteriole-to-venule ratio may be associated with AMD severity, with narrower arterioles seen in the eyes with geographic atrophy, suggesting a role of the retinal vasculature in AMD pathophysiology. This trial is registered with ClinicalTrials.gov Identifier: NCT00000145.

## 1. Introduction

Age-related macular degeneration (AMD) is a multifactorial, blinding disease of old age, and its pathogenesis has been attributed to various factors, including oxidative stress, immune dysregulation, and lipid deposition. Studies using optical coherence tomography (OCT) and OCT angiography (OCT-A) have also suggested a hemodynamic contribution to AMD pathogenesis, with evidence of choroidal thinning, choroidal vascular index changes, or choriocapillaris flow deficits across various stages of the disease [1–13].

Although AMD affects the outer retina and retinal pigment epithelium (RPE), which are primarily supplied by the choroid, some recent studies suggest that retinal vasculature may also be involved. Retinal vessel density as measured on OCT-A is reduced in the eyes with exudative AMD, compared with nonexudative AMD, and is unaffected by antivascular endothelial growth factor (anti-VEGF) treatments [14]. The presence of a cilioretinal artery, which provides ancillary blood flow to inner retinal circulation, also appears to be protective against choroidal neovascularization (CNV), but not central geographic atrophy (CGA) [15]. These findings suggest that impairment in retinal vascular perfusion could also play a role in CNV development in the eyes with AMD, where pathologic features such as soft drusen or Bruch's membrane thickening may hinder oxygen transport from the choroid and disrupt the balance between retinal and choroidal vascular supply. Therefore, determining the relationship between retinal vascular caliber and AMD status could provide insight into whether differences in retinal circulation may impact AMD severity or risk for developing CNV or CGA.

In this study, we further explore the role of the retinal vasculature in AMD by investigating retinal vessel parameters including retinal arterial or venous diameters and arteriole-to-venule ratio (AVR) in fundus photographs from the prospective Age-Related Eye Disease Study (AREDS).

## 2. Methods

### 2.1. Image Dataset

We retrospectively analyzed digitized color fundus photographs obtained from the AREDS through the National Eye Institute's Online Database of Genotypes and Phenotypes (dbGAP accession phs000001, v3.p1.c2) with approval for authorized access [15]. AREDS was originally designed to evaluate the use of oral antioxidants for the treatment of AMD [16]. The study included 11 clinical centers with 4757 patients aged 55–80 years and randomized participants into 1 of 4 treatment conditions: placebo, antioxidants (vitamin C, vitamin E, and *β*-carotene), zinc only, and antioxidants plus zinc [17]. The AREDS adhered to the tenets of the Declaration of Helsinki and was conducted before the existence of the Health Insurance Portability and Accountability Act. The AREDS protocol was approved by an independent data and safety monitoring committee and by the institutional review board for each clinical center. Enrolled patients were scheduled for routine 6-month clinical eye examinations, visual acuity assessments, and supplement dispensing and adherence assessments [18]. Written informed consent was obtained from all participants before enrollment in the AREDS study. Standardized stereoscopic 30° color fundus photographs were obtained from each patient annually and at nonannual visits if participants had decreased visual acuity. The study was exempted by the Institutional Review Board at the University of California, Davis, due to the use of deidentified fundus photographs and trial data.

### 2.2. Image Analysis

**Figure 1:**
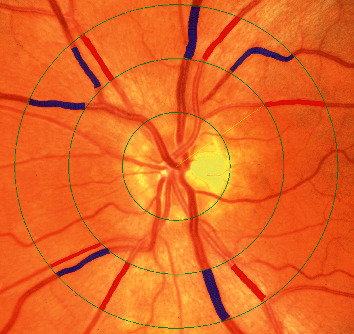
Automated segmentation of retinal vessel diameters from AREDS fundus photographs. The IVAN measurement grid consists of 3 concentric rings with the area of vessel measurement located 0.5 to 1.0-disc diameters away from the optic disc margin. Masked graders correct the automated measurements by adjusting grid centration, manually adding, or deleting vessels, and assigning the appropriate vessel type. Arterioles are displayed in red and venules in blue. Vessels are only measured up until the first branching point within the zone of measurement.

Field 1 fundus photographs, which are centered over the optic disc, from both the left and right eyes of each subject were evaluated by two independent image graders using the semiautomated interactive vessel analysis (IVAN) software from the University of Wisconsin Fundus Photographer Reading Center [19–24]. Each grader manually adjusted the automatic segmentation of the 6 largest venules and arterioles within a concentric ring measuring 0.5–1.0 disc diameters away from the disc center (Figure 1), equivalent to inner and outer diameters of 3600 *μ*m and 4400 *μ*m, which are calibrated to a distance of 4,500 *μ*m measured from the center of the optic disc to the center of the fovea. Manual adjustments were taken to ensure proper grid centration and identification of arterioles and venules. Images with poor visualization due to media opacities or poor image quality or where overlapping arteries and veins precluded clear vessel delineation for segmentation were excluded from the study. Measurements were only taken from vessels up until the first branching within the ring, while any tracings past the branch were manually deleted. These measurements were used to determine the projected central retinal artery equivalent (CRAE), central retinal vein equivalent (CRVE), and the arteriole-to-venule ratio (AVR), which is the ratio of CRAE to CRVE [25]. The Parr–Hubbard–Knudtson formula was used to standardize measurements to enable comparisons between the arterial and venous caliber of different eyes [26]. The 0.5–1.0 disc diameter concentric zone around the optic disc has been established to enable clear distinction between arterioles from arteries while minimizing vessel overlap to allow reliable measurements, based on the work by Parr and colleagues [27–29]. Hubbard et al. extended this formula to include venules [30], which was later refined by Knudtson et al. to adjust for variability in vessel numbers and diameters in this zone [31]. The use of this revised formula has been validated using data from the Beaver Dam Eye Study among other studies [32–34].

### 2.3. Clinical Data

We collected age, sex, smoking status (never, former, or current), and the 9-step AMD severity score of each eye at the baseline and 5-year followup visit. Because our previous study showed that the presence of a cilioretinal artery is associated with lower AMD severity and CNV prevalence, as well as the incidence of CNV at 5 years, we also determined the presence of a cilioretinal artery in each study eye from the corresponding field 2 fundus photographs as previously described [15]. All demographic and clinical data were collected from dbGAP. The clinical criteria for AMD diagnosis, severity, and grading for inclusion in the AREDS study population and the grading protocol used by the Wisconsin Reading Center for classifying AMD severity have been previously reported [17, 18].

### 2.4. Statistical Analysis

Multivariate linear regressions were performed to determine the impact of age, sex, smoking status, and presence or absence of a cilioretinal artery on CRAE, CRVE, and AVR. We also performed univariate regressions to determine which clinical or retinal vascular parameters may be associated with AMD severity (linear regression) or the presence of CNV or CGA (logistic regression), based on the original AREDS reading center gradings. Factors with *P* values of 0.05 or less on univariate regression were selected for a multivariate model to determine which characteristics are independently associated with AMD severity, CNV, or CGA at baseline. For the eyes without advanced AMD (CNV or CGA) at baseline, we used a similar model selection procedure for multivariate regression to determine factors independently associated with a change in 9-step AMD severity score (linear regression) or incident CNV or CGA (logistic regression) at 5 years. All regression analyses were performed with generalized estimating equations to account for two eyes per patient. A *P* value of 0.05 or less was considered to be statistically significant in the multivariate models. Intraclass correlation coefficients (ICCs) were used to determine intergrader agreement of retinal vessel measurements. Statistical analyses were performed using SPSS software (version 25, IBM).

## 3. Results

### 3.1. Clinical Characteristics and Retinal Vessel Parameters

A total of 1190 eyes with field 1 fundus images from 595 patients were available from the online database, of which 18 eyes from 17 patients were excluded as ungradable due to poor visualization, as defined by our exclusion criteria. Among the 594 AREDS participants with 1172 eyes included in our analysis, the mean (±SD) age was 69 ± 5 years (range 55.7–80.2 years), with 55% female, 46.8% former smokers, and 9% current smokers.

**Figure 2:**
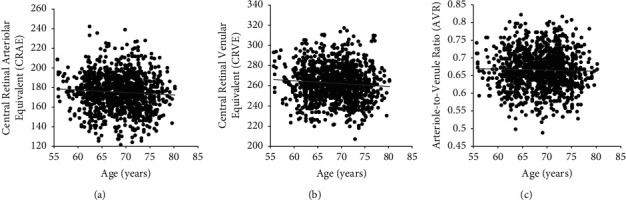
Relationship of retinal vessel parameters with age. Scatterplots showing the relationship of age with (a) Central Retinal Arteriolar Equivalent (CRAE); (b) Central Retinal Venular Equivalent (CRVE); (c) Arteriole-to-Venule Ratio (AVR), with regression trend line.

Mean CRAE was 175.3 ± 18.1 mm, CRVE was 262.5 ± 17.3 mm, and AVR was 0.67 ± 0.05 across all subjects (Table 1). These measurements showed excellent intergrader reliability, with ICC of 0.927 for CRAE, 0.939 for CRVE, and 0.851 for AVR. Both CRAE and CRVE showed a trend toward narrowing with age (Figure 2), consistent with prior studies, although neither reached statistical significance (*P*=0.111 − 0156, Table 2). Additionally, AVR remained stable across the different age groups (Figure 2). Retinal vessel diameters were slightly lower in women compared to men, including both CRAE (*B* = −3.104, *P* < 0.001) and CRVE (*B* = −5.510, *P* < 0.001), but not AVR (*P*=0.547). Current smokers exhibited wider vessel calibers in both arterioles (*B* = 9.455, *P* < 0.001) and venules (*B* = 13.687, *P* < 0.001), with no impact on their ratio (*P*=0.915), compared with former or never smokers (Tables 1 and 2). Interestingly, the eyes with a cilioretinal artery were associated with larger retinal vessels, including CRAE (*B* = 3.467, *P*=0.009) and CRVE (*B* = 2.631, *P*=0.043), even after adjusting for age, sex, and smoking status (Tables 1 and 2). AVR did not appear clearly affected by cilioretinal artery status (*P*=0.096), suggesting that both arterioles and venules are larger by a similar degree in the eyes with this type of vessel. Together, our results indicate that age, sex, smoking status, and the presence of a cilioretinal artery may all impact retinal vessel calibers in our study cohort.

### 3.2. Association of Retinal Vessel Parameters with AMD at Baseline

Among the 1143 eyes with gradable retinal vessel diameters and available baseline AMD severity score, AMD severity was associated with older age (*B* = 0.102, *P* < 0.001), current smokers (*B* = 1.071, *P*=0.017), absence of a cilioretinal artery (*B* = −1.009, *P* < 0.001), and lower AVR (*B* = −4.853, *P*=0.027), but not CRAE (*P*=0.240) or CRVE (*P*=0.500), on univariate analysis (Table 3). These findings were sustained in multivariate regression, with more severe AMD seen in older patients (*B* = 0.104, *P* < 0.001), current smokers (*B* = 1.109, *P*=0.012), and the eyes without a cilioretinal artery (*B* = −0.909, *P*=0.001) and lower AVR (*B* = −4.592, *P*=0.034). Thus, our study showed that lower AVR showed an independent association with greater AMD severity.

Next, we investigated whether retinal vessel parameters may specifically impact CNV or CGA prevalence. Although CNV presence was associated with both older age (*B* = 0.052, *P*=0.003) and absence of a cilioretinal artery (*B* = -0.720, P = 0.009) on multivariate regression as previously reported [15], retinal vascular calibers showed no significant association (*P*=0.269 − 0.821, Table 3). Interestingly, CGA was not associated with age, sex, smoking status, or cilioretinal artery status, but was associated with arterial vessel narrowing (*B* = −0.026, *P*=0.007) and lower AVR (*B* = −5.794, *P*=0.046).

### 3.3. Association of Retinal Vessel Parameters with AMD Progression

From the 820 eyes with gradable retinal vessel calibers and no CNV or CGA at baseline, we found that none of the clinical characteristics or retinal vessel parameters were associated with the change in AMD severity score at 5 years (*P*=0.089 − 0.948). The only factor associated with CNV incidence was older age (*B* = 0.058, *P*=0.023), while the only factor associated with CGA incidence was current smokers (*B* = 0.962, *P*=0.026) as given in Table 4. Neither retinal arterial nor venous calibers impacted AMD progression. Together, our data suggest that AMD severity may be associated with a lower AVR and that CGA may be associated with arteriolar narrowing, but retinal vessel diameters did not appear to predict 5-year AMD progression.

## 4. Discussion

Many past studies have evaluated retinal vessel calibers across various cardiovascular conditions such as hypertension and diabetes [35–37]. A few reports suggested possible enlargement of arteriolar and/or venous diameters in small cohorts of AIDS patients with AMD [38]or Asians with early AMD [23, 39]. However, these population-based studies had only a small number of patients with actual AMD, included predominantly Asian populations or uniquely diseased cohorts, and did not distinguish AMD type or stratify patients based on AMD severity. Here, we present a rigorous analysis of retinal vascular caliber measurements in patients from the large, prospective AREDS, which includes a much larger cohort of AMD patients from a predominantly white, Caucasian population that better reflects the demographics of AMD patients in the U.S. In this study, we found an inverse relationship between AVR and AMD severity and arteriolar attenuation in the eyes with CGA, but no clear independent relationship with CNV. Our study also confirmed our prior finding demonstrating a protective role of the cilioretinal artery against CNV and revealed an interesting relationship between retinal vessel diameters and the presence of a cilioretinal artery. Together, our data suggest that the retinal vasculature may play a role in AMD pathophysiology.

The finding that lower AVR may be associated with worse AMD severity is consistent with our hypothesis that impairment of choroidal perfusion by the presence of soft drusen, basal linear deposits, basal laminar deposits, and/or Bruch's membrane thickening could increase the dependence on retinal circulation [40–43]. This is consistent with some OCT-A studies demonstrating reduced retinal vascular densities in the eyes with early or intermediate AMD [44–46]. We also observed narrower arteriolar diameters and lower AVR in the eyes with CGA, which also reflects OCT-A studies showing lower retinal vessel density in central but not noncentral GA [14]. These associations do not distinguish whether the vascular narrowing is a cause or result of GA, but the fact that none of the retinal vascular parameters predicted incident CGA at 5 years suggests that the narrow vessels could be a consequence of retinal atrophy, as seen in inherited retinal degenerations. Several studies have also found an association between larger retinal arterioles [23], venules [39], or both [38] and early or intermediate AMD. Although our study focused on late AMD, our finding that lower AVR is linked to greater AMD severity could reconcile the findings from other smaller studies. Interestingly, we did not detect an association between retinal vascular caliber with CNV prevalence or incidence as we had hypothesized, although the measurement of large caliber arterioles and venules may not be as sensitive as measuring the density of finer capillary vessels on OCT-A [14].

Our current study confirmed several demographic and clinical factors associated with retinal vascular diameters. We found that females exhibited narrower arterioles as reported in the cardiovascular health study [47] and narrower venules as reported in the Blue Mountain Eye Study [48]. Our findings contrasted with the Multiethnic Study of Atherosclerosis (MESA) [49] where females had larger arterioles and no difference in venule diameters, possibly due to differences in measurement methodology and difference in the study population, as the AREDS cohort was predominantly white/Caucasian, while MESA included more blacks, Hispanics, and Chinese subjects. Gender differences in vessel calibers have been attributed to estrogens, although the proposed mechanism is conflicting in the literature [35]. We also found that current smokers showed larger CRAE and CRVE compared to past and never smokers, consistent with data from the Rotterdam Study [35] and Adult Health Study (AHS) of Japanese atomic bomb survivors [50]. Our analysis showed a trend toward narrower retinal vessels with increased age as reported in these studies, but our data did not reach a statistical significance, likely due to the older and narrower age range of AREDS participants compared with other studies.

Interestingly, we found that the presence of a cilioretinal artery was independently associated with larger CRAE and CRVE values, even after adjusting for age, sex, and smoking history. Because the cilioretinal artery arises from the posterior ciliary artery or peripapillary choroid and is distinct from the retinal vasculature which arises from the central retinal and ophthalmic arteries, the difference in hemodynamics could explain the difference in retinal vessel caliber. Our current study also confirmed the protective effect of the cilioretinal artery against CNV as demonstrated in prior studies [15].

Retinal vascular calibers did not appear to affect AMD progression or incidence of CNV or CGA at 5 years in this study. However, neither age nor smoking status appeared to be associated also, likely due to the smaller number of these events in the subset of the eyes that had available field 1 fundus photographs and no CNV or CGA at baseline. Our study was also limited by the reliance on AMD severity scores as determined from the original reading center for the AREDS, rather than a more refined or granular assessment of AMD features such as noncentral or nascent GA [51] or those determined using OCT. Retinal vessel calibers measured from fundus photographs may not be an accurate measure of vessel lumen diameter or blood flow, and peripapillary vessel diameters may also be an inaccurate indicator of hemodynamics in the macula as retinal capillary density measured on fluorescein angiography or OCT-A. Also, we calibrated fundus images based on the distance between the optic disc and foveal center which may not account for individuals' variability in ocular anatomy, although AVR is a dimensionless ratio which mitigates variations in image magnification. Nevertheless, the strengths of our study include the large size of the cohort, standardized protocol for image capture, and use of reading center determination of AMD severity scores from a prospective study. We also employed dual masked graders with excellent intergrader agreement and confirmed multiple known factors associated with retinal vascular diameters.

In conclusion, our study found that a lower AVR may be associated with worse AMD severity and that arteriolar narrowing may be seen in eyes with CGA, after adjusting for age, sex, smoking status, and the presence of a cilioretinal artery. Thus, changes in retinal circulation may be associated with AMD pathophysiology, and future studies may provide greater insight into its role in this condition.

## Figures and Tables

**Table 1:** Retinal vessel diameters based on patient demographic and clinical characteristics.

	No. of patients	No. of eyes	CRAE (mean ± SD)	CRVE (mean ± SD)	AVR (mean ± SD)
Total	594	1172	175.3 ± 18.1	262.5 ± 17.3	0.67 ± 0.05

Sex					

Male	270	538	177.0 ± 16.2	265.5 ± 15.0	0.66 ± 0.05
Female	324	634	173.8 ± 19.4	260.0 ± 18.6	0.67 ± 0.06

Smoking status					

Never	261	518	174.7 ± 17.2	260.4 ± 16.8	0.67 ± 0.05
Former	279	548	174.1 ± 18.2	262.1 ± 16.4	0.66 ± 0.05
Current	54	106	184.1 ± 18.9	275.0 ± 19.0	0.67 ± 0.05

Cilioretinal artery					

Present	206	228	174.8 ± 18.3	262.2 ± 17.3	0.67 ± 0.05
Absent	551	893	177.8 ± 17.7	264.2 ± 17.3	0.66 ± 0.05

CRAE, central retinal arteriolar equivalent; CRVE, central retinal venular equivalent; AVR, arteriole-to-venule ratio; SD, standard deviation.

**Table 2:** Relationship of retinal vessel diameters with clinical characteristics.

Factor	Category/increment	CRAE			CRVE			AVR		
		*B*	95% CI	*P* value	*B*	95% CI	*P* value	*B*	95% CI	*P* value
Age	1 year	−0.187	−0.446, 0.072	0.156	−0.214	−0.478, 0.049	0.111	0.000	−0.001, 0.001	0.588
Sex	Female vs. male	−3.104	−4.768, −1.441	<0.001	−5.510	−7.044, −3.976	<0.001	0.002	−0.003, 0.007	0.499
Smoking status	Never	Ref			Ref			Ref		
	Former	−0.299	−3.036, 2.437	0.830	1.481	−1.104, 4.066	0.262	−0.005	−0.013, 0.002	0.169
	Current	9.455	4.382, 14.528	<0.001	13.687	8.449, 18.926	<0.001	0.001	−0.014, 0.015	0.915
Cilioretinal artery	Present vs. absent	3.467	0.868, 6.066	0.009	2.631	0.085, 5.178	0.043	0.007	−0.001, 0.015	0.096

CRAE, central retinal arteriolar equivalent; CRVE, central retinal venular equivalent; AVR, arteriole-to-venule ratio (AVR); B, beta coefficient; CI, confidence interval.

**Table 3:** Retinal vascular parameters associated with AMD severity.

Factor	Category/incremen*t*	AMD severity score (*n* = 1143)			Presence of CNV (*n* = 139)			Presence of CGA (*n* = 21)		
		*B*	95% CI	*P* value	*B*	95% CI	*P* value	*B*	95% CI	*P* value
Univariate										

Age	1 year	0.102	0.049, 0.155	<0.001	0.052	0.017, 0.087	0.003	−0.023	−0.125, 0.078	0.651
Sex	Female vs. male	−0.068	−0.376, 0.240	0.663	−0.018	−0.401, 0.364	0.926	−0.267	−1.134, 0.601	0.547
Smoking status	Never	Ref			Ref			Ref		
	Former	0.365	−0.175, 0.905	0.185	−0.064	−0.419, 0.291	0.723	−	−1.041, 0.760	0.759
	Current	1.071	0.189, 1.953	0.017	0.281	−0.261, 0.822	0.310	−0.018	−1.535, 1.500	0.982
Cilioretinal artery	Present vs. absent	−1.009	−1.546, −0.473	<0.001	−0.721	−1.257, −0.186	0.008	−0.081	−1.169, 1.006	0.883
CRAE	1 mm	−0.008	−0.022, 0.006	0.240	−0.001	−0.011, 0.009	0.821	−0.026	−0.045, −0.007	0.007
CRVE	1 mm	0.005	−0.010, 0.020	0.500	0.005	−0.005, 0.015	0.343	−0.022	−0.051, 0.007	0.132
AVR	—	−4.853	−9.148, 0.559	0.027	−1.940	−5.381, 1.500	0.269	−5.794	−11.48, −0.112	0.046

Multivariate										

Age	1 year	0.104	0.053, 0.155	<0.001	0.052	0.017, 0.087	0.003			
Smoking status	Never	Ref								
	Former	0.299	−0.242, 0.841	0.278						
	Current	1.109	0.246, 1.971	0.012						
Cilioretinal artery	Present vs. absent	−0.909	−1.439, −0.378	0.001	−0.720	−1.256, −0.183	0.009			
AVR		−4.592	−8.842, −0.342	0.034						

AMD, age-related macular degeneration; CNV, choroidal neovascularization; CGA, central geographic atrophy; B, beta coefficient; CI, confidence interval; CRAE, central retinal arteriolar equivalent; CRVE, central retinal venular equivalent; AVR, arteriole-to-venule ratio.

**Table 4:** Retinal vascular parameters associated with AMD progression after 5 years.

Factor	Category/increment	Change in AMD severity score (*n* = 820)			Progression to CNV (*n* = 95)			Progression to CGA (*n* = 64)		
		*B*	95% CI	*P* value	*B*	95% CI	*P* value	*B*	95% CI	*P* value
Age	1 year	0.005	−0.013, 0.023	0.574	0.058	0.008, 0.108	0.023	0.056	−0.008, 0.120	0.087
Sex	Female vs. male	−0.053	−0.201, 0.095	0.486	0.116	−0.288, 0.521	0.573	0.019	−0.439, 0.477	0.935

Smoking status	Never	Ref			Ref			Ref		
	Former	0.138	−0.044, 0.319	0.137	0.344	−0.128, 0.816	0.154	0.175	−0.440, 0.790	0.577
	Current	0.289	−0.044, 0.622	0.089	0.558	−0.202, 1.319	0.150	0.962	0.114, 1.811	0.026
Cilioretinal artery	Present vs. absent	−0.089	−0.272, 0.094	0.339	−0.157	−0.684, 0.370	0.370	−0.083	−0.730, 0.563	0.801
CRAE	1 mm	0.001	−0.003, 0.006	0.527	−0.007	−0.019, 0.006	0.280	−0.002	−0.019, 0.015	0.795
CRVE	1 mm	0.003	−0.002, 0.007	0.288	−0.003	−0.016, 0.010	0.685	0.004	−0.011, 0.020	0.576
AVR	—	0.049	−1.409, 1.506	0.948	−2.350	−6.443, 1.742	0.260	−1.991	−7.799, 3.817	0.502

AMD, age-related macular degeneration; CNV, choroidal neovascularization; CGA, central geographic atrophy; B, beta coefficient; CI, confidence interval; CRAE, central retinal arteriolar equivalent; CRVE, central retinal venular equivalent; AVR, arteriole-to-venule ratio.

## Data Availability

The data used to support this study are available from the corresponding author upon request.
